# Unveiling the psychological network of work alienation among nursing interns: A resource conservation perspective and network analysis

**DOI:** 10.1371/journal.pone.0351867

**Published:** 2026-06-17

**Authors:** Yang Guo, Xixi Huang, Chengguo Guan, Ruonan Wang, Jie Yao, Luying Yang, Sha Li

**Affiliations:** 1 Department of Nursing, School of Medicine, Shaanxi Institute of International Trade & Commerce, Xianyang, Shaanxi, China; 2 Shaanixi Open University School of Nursing and Health Sciences, Shaanxi Open University, Xi’an, Shaanxi, China; 3 Department of Nursing, Medical College, Xijing University, Xi’an, Shaanxi, China; 4 Nursing College, Shaanxi University of Chinese Medicine, Xi’an, Shaanxi, China; 5 Nursing Department, The Second Affiliated Hospital of Shaanxi University of Chinese Medicine, Xi’an, Shaanxi, China; Alexandria University Faculty of Nursing, EGYPT

## Abstract

**Background:**

Clinical internship is critical for nursing students, but heavy workload and emotional demands increase the risk of work alienation. Traditional linear models fail to capture complex interrelationships among psychological factors.

**Objective:**

To apply psychological network analysis to explore the associative structure of work alienation in nursing interns, identifying central and bridge nodes to generate hypothesis‑generating intervention priorities.

**Methods:**

A cross-sectional survey was employed. Nursing interns from four tertiary hospitals in the Guanzhong region of Shaanxi Province were recruited via convenience sampling from January to August 2025. Data were collected using the Nurse Work Alienation Scale, Compassion Fatigue Scale, Moral Distress Scale, Ethical Sensitivity Questionnaire for Nursing Students, and NASA Task Load Index. A total of 934 valid responses were obtained. A regularized partial correlation network model was estimated using the EBICglasso method (γ = 0.5). Node strength and bridge strength were calculated, and stability was assessed via bootstrap.

**Results:**

Node strength analysis identified personal responsibility (1.19), burnout (1.18), and failure to maintain patient’s best interest (1.13) as the three most central nodes. Bridge strength analysis revealed secondary traumatic stress (STS) as the strongest bridge (0.43, 95% CI [0.31, 0.55]), followed by perceived workload (0.38) and self‑evaluation (0.38). Subgroup network comparisons showed no significant structural differences by gender, age, or education (all p > 0.05). Stability analysis confirmed good robustness for centrality estimates.

**Conclusion:**

Psychological network analysis mapped the associative structure of work alienation, identifying personal responsibility, burnout, and STS as key hub and bridge nodes. These findings offer hypothesis‑generating targets for future interventions (e.g., trauma‑informed care, workload management, self‑efficacy enhancement), pending validation in longitudinal studies.

## Introduction

Clinical internship is a critical transitional phase for nursing students, moving from learners to clinical practitioners, and a key component of their professional socialization process. However, the high-intensity job demands, complex nursing tasks, and interpersonal and emotional pressures during internships place student nurses at high risk for work alienation [1,2]. As a negative psychological experience, work alienation manifests as feelings of powerlessness, meaninglessness, and emotional detachment towards nursing work. It not only undermines students’learning motivation and professional identity but may also trigger job burnout, reduce retention intention, and pose a potential threat to the sustainable development of the nursing workforce [1,2]. Previous research has predominantly focused on the direct association between external factors, such as task load, and alienation, often overlooking the heterogeneity of individual psychological responses under similar stressful conditions [3,4]. Consequently, it has been difficult to fully unveil the internal mechanisms underlying the formation of alienation. Explaining the intensity and variation of the alienation experience solely from the perspective of external load is clearly insufficient. There is an urgent need to introduce an analytical framework capable of dissecting the complex interactions among variables to deeply explore its pathways of influence from a systemic perspective [4].

The Conservation of Resources Theory posits that individuals have a fundamental motivation to preserve, maintain, and acquire key resources [5,6]. When confronted with a stressful environment that may lead to resource loss, they develop strong psychological defenses [5]. Consequently, work alienation can be understood as a critical psychological outcome arising from chronic resource depletion experienced by nursing interns in clinical settings that a defensive withdrawal adopted by the individual to protect themselves amidst continuous resource erosion [7]. According to this theory, the process of resource depletion begins with persistent environmental demands [5,6]. In clinical settings, the most direct manifestation of such demand is task load, which constitutes a sustained external stressor that consumes the cognitive and physical resources of nursing interns [7].

However, resource depletion is not a homogeneous process. It manifests through distinct psychological pathways. When nursing interns are unable to uphold the ethical principles they value due to clinical constraints, they experience a specific form of resource loss, leading to moral distress [8,9]. This signifies the impairment of core resources such as moral integrity and professional values. Simultaneously, frequent exposure to patient suffering and the sustained demand for emotional support in clinical environments may result in another form of resource exhaustion, known as compassion fatigue, which represents the depletion of emotional and empathetic resources [9,10]. Furthermore, susceptibility to resource depletion varies among individuals. One key trait influencing this variability is ethical sensitivity, which refers to an individual’s inherent tendency to perceive and interpret ethical issues and the suffering of others. This trait determines the acuity with which individuals identify threats to their resources. Those with higher ethical sensitivity may perceive the threat of resource loss earlier and more intensely under similar high-pressure conditions, thereby potentially altering the entire dynamic pathway of resource depletion [8,10].

Therefore, task load, moral distress, compassion fatigue, ethical sensitivity, and work alienation together theoretically constitute a dynamically interconnected psychological system [1,7,8]. While traditional structural equation modeling or regression analysis can examine the net effects among variables and preset mediating/moderating pathways, their assumptions of linear and unidirectional causality may fail to adequately capture the dynamic, bidirectional, and nonlinear interactions among these psychological variables in clinical practice [7,11]. For example, work alienation may not only be the endpoint of resource depletion but could also reciprocally undermine an individual’s motivation and ability to cope with task load, creating a vicious cycle [12]. Compassion fatigue may intensify the perception of moral distress, which in turn may deepen emotional detachment [13]. To characterize the true configuration of this complex system, this study introduces network analysis [11]. Unlike traditional linear models that presuppose unidirectional causal pathways and require pre‑specified mediator/moderator relationships, network analysis offers two unique advantages: Firstly, it visualizes the complex, bidirectional interconnections among all variables simultaneously without imposing a priori assumptions [14]. Secondly, it quantifies the relative importance of each variable (centrality) and each cross‑construct connection (bridge strength) within the overall system [15]. This approach allows us to move beyond merely confirming whether variables are associated to uncovering the specific structural architecture of resource depletion, potentially identifying novel intervention targets that are not apparent from simple pairwise correlations.

This approach treats the aforementioned psychological variables as an interrelated system, where variables serve as “nodes” and the statistical associations between them (after controlling for all other variables) constitute “edges”. This method allows us to visualize and quantify the structural features of the entire resource depletion system, such as whether direct, strong feedback loops exist between task load, moral distress, and compassion fatigue [16], and whether individuals with high versus low ethical sensitivity exhibit significantly different overall psychological network structures [11]. By constructing and comparing psychological networks, this study aims to systematically uncover the complex mechanisms and boundary conditions of dynamic resource depletion among nursing interns in high-pressure environments. This not only deepens the theoretical application of the Conservation of Resources Theory in nursing education by shifting the research perspective from a singular cause-effect chain to a system-interaction view but also allows for the identification of core nodes and strong connections within the network. This implies that interventions can be prioritized toward the most influential psychological processes, thereby efficiently dismantling the negative network that sustains work alienation and promoting the professional mental health and steady career growth of nursing interns.

## Research methods

### Study design

This study employed a cross-sectional survey design.

### Study participants

The participants were enrolled undergraduate nursing students from the Guanzhong region of Shaanxi Province. (1) Inclusion criteria: ① Nursing interns in the Guanzhong region who had completed at least three months of clinical internship; ② Nursing interns who voluntarily participated in this survey and were able to complete the questionnaire truthfully. (2) Exclusion criteria: ① Nursing interns with self‑reported, physician‑diagnosed psychological disorders (e.g., major depressive disorder, generalized anxiety disorder) or severe mental illnesses (e.g., bipolar disorder, schizophrenia). No standardized diagnostic scales were administered; exclusion was based solely on participant disclosure of a formal diagnosis, which may introduce selection bias. ② Nursing interns who did not complete their internship due to leave, health issues, or other reasons; ③ Nursing interns who provided incomplete information or obviously erroneous responses.

Based on Kendall’s sample estimation method, the required sample size should be 5–10 times the maximum number of variables [17]. Considering potential errors from invalid questionnaires and sample attrition, a 20% attrition rate was accounted for. Therefore, the final sample size for this survey was determined to be 594–1188 participants.

### Data collection process

Phase 1: Establishment of the Research Team and Literature Review

(1) A research team was formed, consisting of 3 nursing faculty members and 4 head nurses from tertiary hospitals. Based on an extensive literature review and considering the current working conditions of nursing interns in Shaanxi Province, the research topic and content were determined.
(2) Selection of research tools: Based on the literature review, relevant research instruments were searched according to the study variables. Appropriate scales were selected based on the study objectives and the applicability of the tools. Finally, a self-developed questionnaire was combined with the scales used for each variable to form the final questionnaire.

Phase 2: Questionnaire Distribution and Collection

(1) Pilot survey phase: A convenience sampling method was used to select 30 nursing interns from a hospital in Xianyang City for the pilot survey. Before conducting the survey, the researchers first applied to the hospital’s nursing department director for permission to conduct this pilot survey. The drafted questionnaire was submitted to the hospital’s nursing department for review. After approval, the pilot survey was initiated. Issues identified during the pilot survey process were addressed, such as deleting or modifying unreasonable items, and the survey protocol was revised to ensure the smooth progress of the formal survey.
(2) Formal survey phase: Based on the research objectives, a convenience sampling method was employed to select nursing interns from four tertiary hospitals (all hospitals were general hospitals) in the Guanzhong region of Shaanxi Province. The student sources covered the entire Guanzhong region, ensuring the sample size had a certain degree of universality and representativeness. Participants were then screened according to the inclusion and exclusion criteria. Before distributing the questionnaires, the nursing department heads of the internship hospitals were contacted, and the survey questionnaire was sent to each hospital via email, WeChat, or QQ for review. After approval, the questionnaire was distributed online using the electronic platform “Questionnaire Star”. An informed consent form was included in the questionnaire. Participants were informed that their participation was voluntary and based on informed consent. The questionnaire was anonymous and confidential, and the collected data were used solely for academic research. Additionally, the 30 students who had participated in the pilot survey were excluded.
(3) Questionnaire collection: After approval by the nursing departments of the surveyed hospitals, the questionnaire was distributed online via the electronic platform “Questionnaire Star”. Before filling out the questionnaire, participants were required to read a detailed digital informed consent form, which included the study’s purpose, the voluntary nature of participation, anonymity and confidentiality measures, data storage policies, and the right to withdraw at any time without penalty. Participants could only proceed to the questionnaire after explicitly agreeing by checking the “I agree to participate” option. The survey questionnaire consisted of six parts: the “Demographic Information Form”, the “Nurse Work Alienation Scale”, the “Compassion Fatigue Scale”, the “Moral Distress Scale” the “Ethical Sensitivity Scale” and the “NASA Task Load Index”. The questionnaires were collected from January to August 2025. A total of 1012 questionnaires were received, of which 78 were invalid and thus excluded, resulting in 934 valid questionnaires retained. The effective response rate was 92.29%. This study was approved by the Medical Ethics Committee of the School of Medicine of Shaanxi Institute of International Trade & Commerce (Ethics Approval No.: HLX-20241223) and was conducted in accordance with the Declaration of Helsinki.

### Research instruments

(1) Demographic Information Form: Designed by the researchers, this general information form included 12 items such as gender, age, education level, ethnicity, religion, place of origin, attitude towards the nursing profession, and whether the intern worked night shifts.
(2) Nurse Work Alienation Scale: The Work Alienation Scale developed by Ren Xiaojing was used to assess the level of work alienation among nurses or nursing students [18]. The scale consists of 12 items across three dimensions: helplessness (4 items), resignation (4 items), and meaninglessness (4 items). A 5-point Likert scale was used, ranging from “strongly disagree” to “strongly agree”. Higher total scores indicate more severe work alienation. The scale’s Cronbach’s alpha coefficient was 0.890 [19], demonstrating good reliability and validity. In this study, its Cronbach’s alpha coefficient was 0.910.
(3) Compassion Fatigue Scale: The nurse version of the Compassion Fatigue Scale revised by Chen Huaying et al. [20] was used. This scale includes 3 dimensions and 30 items, assessed using a 5-point Likert scale (1–5 points). The total score ranges from 30 to 150, with higher scores indicating a higher level of compassion fatigue. In this study, the scale’s Cronbach’s α coefficient was 0.844 [20].
(4) Moral Distress Scale (MDS): The Nurse Moral Distress Scale was originally developed by Corley et al. [21] in 1995 and later revised by Hamric et al. to 21 items, with a Cronbach’s α coefficient of 0.890. Sun Xia [22] translated and adapted it into Chinese to measure moral distress among nurses. The Chinese version has a Cronbach’s α coefficient of 0.879. The scale consists of 22 items across four dimensions: personal responsibility (8 items), failure to uphold the patient’s best interest (5 items), value conflict (6 items), and harm to the patient (3 items). A 5-point Likert scale is used: frequency of occurrence ranges from “never” to “very frequent,” scored 0–4, and the degree of distress ranges from “none” to “severe,” scored 0–4. The score for each item is the product of its frequency and distress scores. The total score is the sum of all item scores, ranging from 0 to 352, with higher scores indicating a higher level of moral distress. In this study, its Cronbach’s α coefficient was 0.859.
(5) Chinese Version of the Ethical Sensitivity Questionnaire for Nursing Students (ESQ-NS): This scale was translated and adapted into Chinese by Wang Shengnan et al. [23] It includes three dimensions: respect for persons, distributive fairness, and protection of patient privacy, comprising 11 items in total. The content validity for individual items ranged from 0.833 to 1.000, and the scale-level content validity was 0.933. The overall Cronbach’s α coefficient for the scale was 0.926, with sub-dimension coefficients ranging from 0.803 to 0.931. The split-half reliability for the total scale was 0.870, and for the sub-dimensions, it ranged from 0.804 to 0.912. This scale is suitable for assessing the ethical sensitivity level of Chinese nursing students. In this study, its Cronbach’s α coefficient was 0.803.
(6) NASA Task Load Index (NASA-TLX) Scale: The NASA-TLX scale was developed by the National Aeronautics and Space Administration (NASA) in 1988. The Chinese version was developed by Liang Liling et al. [24]. The scale consists of 6 items across two dimensions: perceived workload and self-evaluation. Each item is scored on a scale of 0–20, and the total score ranges from 0 to 120, with higher scores indicating a higher perceived workload. The self-evaluation items are reverse-scored, meaning that a more perfect self-evaluation corresponds to a lower task load. The scale has a Cronbach’s α coefficient of 0.832, demonstrating good reliability and validity. In this study, its Cronbach’s α coefficient was 0.717.

### Statistical analysis

This study employed network analysis to examine the associative structure among the subscales of compassion fatigue, moral distress, ethical sensitivity, work alienation, and workload. All statistical analyses were conducted in the R.

(1) Prior to formal analysis, data were first organized and standardized. All subscale variables from the original questionnaire were uniformly renamed with concise English abbreviations (e.g., CSat, Burnout, STS) to facilitate subsequent modeling, visualization, and result presentation. This study did not include data at the individual item level; only the scores of each subscale were used as analytical variables. Samples with missing values on any subscale were excluded to ensure network estimation was based on complete data. Subsequently, descriptive statistics, including mean, standard deviation, skewness, and kurtosis, were calculated for each subscale to summarize the basic distributional characteristics of the variables. The descriptive statistics were computed based on the raw scale scores and are presented in tabular form.
(2) As the subscales were derived from different instruments with varying scoring ranges and units, all subscale scores were standardized (converted to z‑scores) for the network analysis phase. This procedure facilitates the comparison of association strengths between variables on a common scale and prevents variables with larger numerical ranges from unduly influencing the network structure. The network structure was estimated using a regularized partial correlation‑based method. Nodes represent the subscales, and edges represent the conditional correlations between node pairs after controlling for all other variables. The network was estimated using Spearman correlation coefficients and regularized via the EBICglasso method with a hyperparameter gamma (γ) set to 0.5, a common default that balances model fit and parsimony in psychological network studies. Subscale‑level aggregation was chosen over item‑level analysis to maintain a stable ratio of parameters to sample size (934 valid cases for 15 nodes) and to improve the reliability of each node as a construct indicator, consistent with prior network studies on similar constructs. To aid interpretation, nodes were grouped according to their parent scale and distinguished by a consistent color scheme in the network visualization.
(3) Based on the overall network structure, node strength was further calculated to assess the overall connectedness of each subscale within the network, while bridge strength was computed to identify subscales that serve as connectors between different scale constructs. Finally, the stability of the network was assessed using the bootstrap method (case‑dropping stability analysis), to test the robustness of the strength and bridge strength indicators under different sample conditions.
(4) Common Method Bias (CMB): Given that all data were collected via self‑report scales at a single time point, CMB is a potential concern. Procedural remedies were implemented during the survey design, including: ① Ensuring anonymity and confidentiality. ② Using reverse‑coded items within scales. ③ Separating scale blocks visually. Statistically, Harman’s one‑factor test was conducted on all scale items.

## Results

### CMB results

The unrotated factor solution explained 28.6% of the total variance, which is below the recommended threshold of 40%, suggesting that CMB is unlikely to severely distort the correlational results.

### Participant characteristics

Of the 934 valid participants, 786 (84.2%) were female, and the mean age was (21.4 ± 1.2) years. In terms of education level, 834 undergraduate nursing students (89.3%). Regarding ethnicity, 887 (95.0%) were Han Chinese. A total of 80 (8.6%) reported a religious belief. For place of origin, 730 (78.2%) were from rural areas, and 204 (21.8%) from urban cities. Attitude towards the nursing profession: 551 (59.0%) reported “like”, 383 (41.0%) “it not”. Among the sample, 728 (77.9%) worked night shifts. Other details shown in supplementary materials S1 Table.

### Basic distributional characteristics (mean, standard deviation, skewness, and kurtosis) of the subscales

Overall, significant differences were observed across the subscales in terms of score levels and dispersion, reflecting variations in their origin and scoring methods. Among the compassion fatigue-related subscales, Compassion Satisfaction, Burnout, and Secondary Traumatic Stress showed similar mean scores, with standard deviations around 7. Their distributions exhibited mild right-skewed or approximately symmetrical shapes, and their kurtosis values fell within an acceptable range. The moral distress-related subscales (Personal Responsibility, Fail to Maintain Best Interest, Value Conflict, Harm to Patient) demonstrated notably larger standard deviations, along with higher skewness and kurtosis compared to other subscales. This suggests that their score distributions were more concentrated in the lower range while also displaying a certain degree of long-tailed characteristics. The ethical sensitivity subscales (Respect for Persons, Distributive Fairness, Patient Privacy) showed relatively stable distributions, with skewness close to 0 and kurtosis negative or near 0, indicating relatively symmetrical score distributions and a low level of outliers. Among the work alienation subscales, Helplessness, Resignation, and Meaninglessness exhibited similar distribution patterns, with small values for both skewness and kurtosis, and no significant distributional abnormalities were observed. Compared to other subscales, Perceived Workload and Self-Evaluation had significantly higher means and standard deviations, reflecting differences in their units of measurement and scoring ranges relative to the other questionnaires (see in Table 1). This difference further justifies the necessity of standardizing all subscale scores prior to network analysis, to prevent variations in numerical ranges from affecting the estimation of the associative structure.

**Table 1:** Basic Distributional Characteristics (Mean, Standard Deviation, Skewness, and Kurtosis) of the Subscales.

Items	mean	sd	skew	kurtosis
Compassion satisfaction	25.897	7.305	0.856	1.237
Burnout	26.415	6.905	0.634	1.211
Secondary traumatic stress	29.506	7.263	0.214	0.494
Personal responsibility	16.233	16.021	1.538	6.286
Fail to maintain best interest	11.742	10.049	1.328	5.317
Value conflict	15.426	12.496	1.099	3.629
Harm to patient	6.813	6.466	1.300	4.030
Respect for persons	14.123	3.540	0.171	−0.094
Distributive fairness	7.292	2.462	0.138	−0.591
Patient privacy	4.845	1.784	0.192	−0.724
Helplessness	10.777	3.714	0.121	−0.142
Resignation	12.497	3.503	−0.244	−0.010
Meaninglessness	10.569	3.862	0.085	−0.358
Perceived workload	301.062	78.581	−0.690	−0.048
Self-evaluation	110.193	49.862	−0.053	−0.580

### Overall network structure and node strength at the subscale level

**Fig 1:**
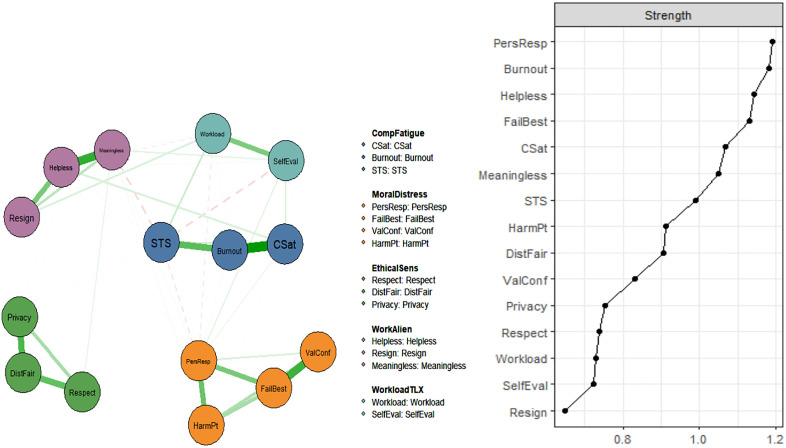
Left panel: Regularized partial correlation network of all 16 subscales. Green solid edges = positive conditional correlations, red dashed edges = negative. Edge thickness = absolute correlation strength. Right panel: Node strength centrality (standardized z‑scores) for each node, with error bars representing 95% bootstrap confidence intervals.

Fig 1 left panel presents the overall network structure estimated based on the standardized subscale scores. In the network, each node represents a subscale, with node colors indicating its parent scale. Edges represent conditional correlations between node pairs after controlling for all other variables. Edge thickness reflects the strength of association, with solid green lines indicating positive correlations and red dashed lines indicating negative correlations. From the overall structure, subscales within the same parent scale generally exhibited strong connections. For example, the compassion fatigue-related subscales (CSat, Burnout, STS) formed a relatively dense local cluster; the moral distress subscales (PersResp, FailBest, ValConf, HarmPt) and the ethical sensitivity subscales (Respect, DistFair, Privacy) also demonstrated notable within-group associations. Connections between different parent scales were relatively weaker but not entirely separate, suggesting that conditional associations still exist to some extent across constructs.

Fig 1 right panel displays the ranked results of node strength for each subscale, reflecting the overall connectedness of each node with all other nodes in the network. The results show clear differences in the level of connectedness among the subscales: PersResp, Burnout, and FailBest exhibited the highest node strength, indicating that these subscales maintained more numerous and stronger conditional associations with other variables. Helpless, CSat, and Meaningless demonstrated moderately high node strength, suggesting they possess a certain breadth of connections within the network but do not occupy the most central positions. Resign, SelfEval, and Workload showed lower node strength, revealing that their direct conditional associations with other subscales were relatively limited. Overall, the node strength results are consistent with the visual pattern observed in the network structure plot, where high-strength nodes are typically located at the intersections of multiple connecting pathways, while low-strength nodes are more distributed towards the periphery of the network.

### Bridge subnetwork structure and bridge strength distribution

**Fig 2:**
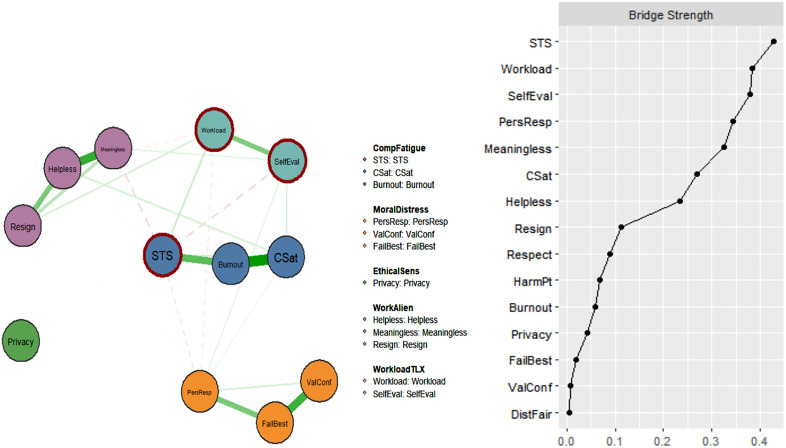
Left panel: Bridge subnetwork identified based on bridge strength filtering (nodes with bridge strength > 0.15). Red borders indicate nodes with high bridge strength. Right panel: Bridge strength centrality (standardized values) for each node, with 95% bootstrap confidence intervals. STS: secondary traumatic stress.

Fig 2 left panel presents the bridge subnetwork identified based on bridge strength filtering. This network, derived from the overall network structure, retains only bridge nodes and their adjacent nodes to highlight the connective pathways between different parent scales. Node colors still represent their respective parent scales, with red borders indicating subdimensions with higher bridge strength. As evident from the bridge subnetwork structure, cross-scale connections are not uniformly distributed but rather concentrated among a few subdimensions. STS, Workload, and SelfEval occupy connecting positions between different scales, maintaining direct associations with multiple constructs. PersResp and Meaninglessness also participate in cross-scale connections, although their connective scope is relatively more localized. In contrast, most ethical sensitivity subdimensions and some moral distress subdimensions demonstrate lower levels of involvement in the bridge subnetwork, manifesting predominantly as within-scale associations.

Fig 2 right panel presents the ranked results of bridge strength for each subdimension, quantifying the degree of connectivity each node maintains across different scales. The results showed that STS exhibited the highest bridge strength (0.43), followed by Workload (0.38), SelfEval (0.38), and PersResp (0.34), indicating that these subscales bear greater associative load in linking different constructs. Meaninglessness and CSat demonstrated moderate bridge strength, suggesting that while they play a role in cross-scale connections, they do not serve as primary pathways. In contrast, ValConf, DistFair, FailBest, and Privacy had bridge strength values close to zero, indicating that their associations are largely confined within their respective scales and contribute minimally to connections between different constructs. Overall, the bridge strength findings align with the visual structure of the bridge subnetwork, confirming that cross-scale connections are predominantly concentrated among a few subdimensions rather than being evenly distributed across all subdimensions.

### Relative importance analysis of core and bridge nodes

To further quantify the relative importance of each subscale within the network structure, this study reports the node strength and bridge strength of all subscales along with their 95% Bootstrap confidence intervals (see Table 2). Regarding node strength, Personal responsibility exhibited the highest value (1.191, 95% CI [1.080, 1.328]), indicating its strongest overall connectivity within the network. Burnout also showed high node strength (1.183, 95% CI [1.106, 1.311]), followed by Helplessness (1.142, 95% CI [1.033, 1.287]), and Fail to maintain best interest (1.131, 95% CI [1.063, 1.236]). These results suggest that subscales such as personal responsibility, burnout, helplessness, and failure to maintain the patient’s best interest occupy relatively core positions in the network, exhibiting strong direct or indirect connections with multiple other psychological or work‑related constructs.

**Table 2:** Node Strength and Bridge Strength for All Subscales with 95% Bootstrap Confidence Intervals.

Subscale	Node Strength	95% CI	Bridge Strength	95% CI
Personal responsibility (PersResp)	1.191	[1.080, 1.328]	0.344	[0.241, 0.475]
Burnout	1.183	[1.106, 1.311]	0.058	[0.015, 0.170]
Helplessness	1.142	[1.033, 1.287]	0.233	[0.139, 0.384]
Fail to maintain best interest (FailBest)	1.131	[1.063, 1.236]	0.020	[0.000, 0.098]
Compassion satisfaction (CSat)	1.069	[0.970, 1.226]	0.269	[0.199, 0.396]
Meaninglessness	1.049	[0.954, 1.191]	0.324	[0.243, 0.468]
STS (Secondary traumatic stress)	0.990	[0.855, 1.188]	0.428	[0.307, 0.621]
Harm to patient (HarmPt)	0.912	[0.845, 1.022]	0.067	[0.026, 0.173]
Distributive fairness (DistFair)	0.905	[0.856, 1.041]	0.006	[0.002, 0.121]
Value conflict (ValConf)	0.831	[0.776, 0.988]	0.008	[0.000, 0.130]
Patient privacy (Privacy)	0.754	[0.697, 0.896]	0.042	[0.008, 0.169]
Respect for persons	0.739	[0.669, 0.863]	0.090	[0.052, 0.195]
Perceived workload (Workload)	0.729	[0.530, 1.000]	0.384	[0.214, 0.640]
Self-evaluation (SelfEval)	0.723	[0.585, 0.903]	0.378	[0.275, 0.537]
Resignation	0.649	[0.564, 0.818]	0.112	[0.051, 0.252]

Node strength and bridge strength are standardized (z‑scored) values. Confidence intervals (95%) were estimated using 1,000 bootstrap resamples with case‑dropping (50% drop) to assess stability. For bridge strength, P ≤ 0.10 indicate negligible cross‑construct connectivity.

The bridge strength results further revealed the role of different subscales in cross‑construct connections. STS exhibited the highest bridge strength (0.428, 95% CI [0.307, 0.621]), indicating that secondary traumatic stress serves as an important bridge node connecting different psychological constructs. This was followed by Perceived workload (0.384, 95% CI [0.214, 0.640]), Self‑evaluation (0.378, 95% CI [0.275, 0.537]), Personal responsibility (0.344, 95% CI [0.241, 0.475]), and Meaninglessness (0.324, 95% CI [0.243, 0.468]). These results suggest that secondary traumatic stress, perceived workload, self‑evaluation, personal responsibility, and meaninglessness may serve as important pathways connecting constructs related to compassion fatigue, moral distress, work alienation, and workload. Together, these findings indicate that subscales such as personal responsibility, burnout, helplessness, and failure to maintain the patient’s best interest occupy relatively core positions in the network, exhibiting strong direct or indirect connections with multiple other psychological or work‑related constructs.

### Stability analysis of network centrality indices

**Fig 3:**
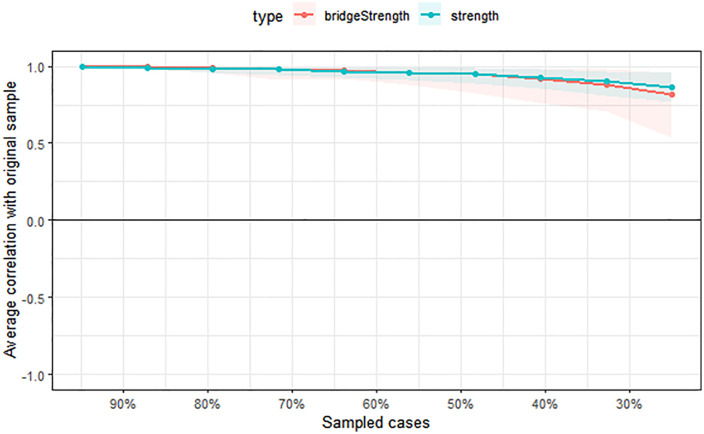
Network stability results based on the case‑dropping bootstrap method. Left panel: stability of node strength; Right panel: stability of bridge strength. The horizontal axis shows the proportion of cases dropped; the vertical axis shows the average correlation with the original centrality indices. Shaded areas represent 95% bootstrap confidence intervals.

Fig 3 presents the network stability results obtained using the case-dropping bootstrap method. The horizontal axis represents the proportion of samples progressively excluded, and the vertical axis represents the average correlation coefficient between the centrality indices under the corresponding sample conditions and those derived from the original sample. The figure illustrates the stability trends for node strength and bridge strength, respectively. The results showed that under low sample dropping proportions, both centrality indices maintained high correlation levels, with coefficients close to 1, indicating good consistency in network structure and centrality rankings under near-complete sample conditions.

As the proportion of excluded samples increased, the correlation coefficients for both strength and bridge strength exhibited a gradual downward trend, although the overall decline was relatively modest. Across most sample dropping intervals, the correlation coefficient for strength remained slightly higher than that for bridge strength, suggesting that the overall connectedness of nodes was relatively more stable under sample reduction. In contrast, bridge strength was more sensitive to sample fluctuations, with its correlation coefficient declining more markedly under high sample dropping proportions; nevertheless, it remained within an acceptable range. Overall, the case-dropping results indicate that node strength and bridge strength indices in this study demonstrated satisfactory stability across different sample conditions, supporting the reliability of the aforementioned network structure and bridge analysis findings.

### Subgroup network analysis results

Based on the sample distribution, psychological network structures were estimated separately for different subgroups using education level, religious belief, ethnicity, gender, and age as grouping variables. Differences in network structure between subgroups were compared using the Network Comparison Test. Furthermore, this study extracted the core node with the highest node strength and the bridge node with the highest bridge strength in each subgroup to identify key variables that may have targeted intervention implications for different populations. The results are presented in Table 3. Overall, the network structure differences between different demographic subgroups did not reach statistical significance. These findings indicate that, in the present sample, the overall psychological network structures across different demographic subgroups were highly consistent, and no significant overall network structure differences were observed.

**Table 3:** Subgroup Network Comparison by Demographic Characteristics.

Subgroup variable	Global strength Group	Difference	Network structure statistic	*p* for network structure	Core node in Group	Bridge node in Group
Education level						
Associate degree (n = 100)	5.949	−1.081	1.081	0.683	FailBest (1.071)	Meaningless (0.239)
Bachelor’s degree (n = 834)	7.030				PersResp (1.196)	STS (0.460)
Religious belief						
Yes (n = 80)	6.125	−0.726	0.726	0.881	PersResp (1.059)	Meaningless (0.287)
No (n = 854)	6.852				Burnout (1.182)	STS (0.407)
Ethnicity						
Han ethnicity (n = 887)	6.854	0.963	0.963	0.896	PersResp (1.192)	STS (0.399)
Ethnic minority (n = 47)	5.890				Privacy (1.072)	SelfEval (0.318)
Gender						
Male (n = 148)	6.924	0.311	0.311	0.904	PersResp (1.196)	STS (0.415)
Female (n = 786)	6.613				FailBest (1.165)	Workload (0.312)
Age						
≤20 (n = 172)	7.045	−0.130	0.130	0.955	HarmPt (1.199)	PersResp (0.358)
>20 (n = 762)	7.175				Burnout (1.207)	Workload (0.497)

PersResp: Personal responsibility; FailBest: Fail to maintain best interest; HarmPt: Harm to patient; STS: Secondary traumatic stress; Workload: Perceived workload; SelfEval: Self-evaluation.

Although the overall network structure differences did not reach statistical significance, certain descriptive differences emerged across subgroups in terms of global network connectivity and key nodes. Regarding global connectivity, the higher education group had a global strength of 7.030, which was higher than that of the lower education group (5.949). The non-religious group exhibited a global strength of 6.852, higher than that of the religious group (6.125). The female group showed a global strength of 6.924, slightly higher than that of the male group (6.613). The global strength values for the younger and older groups were relatively close, at 7.045 and 7.175, respectively. These results suggest that the overall degree of connectivity among psychological variables may differ across demographic subgroups, but such differences mainly manifest as descriptive trends.

## Discussion

### Theoretical interpretation and comparison with prior network studies

This study identified personal responsibility, burnout, and failure to maintain the patient’s best interest as the core variables with the highest node strength in the network, indicating their pivotal roles in the psychological system underlying resource depletion and work alienation among nursing interns [25–27]. While prior research has individually linked moral distress, burnout, and work alienation [25], but the network analysis reveals their relative centrality within a unified system. Critically, it demonstrates that “personal responsibility” and “failure to maintain best interest” are not just correlates but occupy the most influential hub positions, suggesting that interventions targeting ethical dimensions may have broader systemic effects than targeting, for example, task load, which showed lower overall centrality.

From a Conservation of Resources (COR) perspective, this high centrality indicates that “personal responsibility” functions as a keystone resource. When threatened or lost (e.g., by unavoidable clinical constraints), the perceived risk of resource loss amplifies and rapidly spreads to other domains (e.g., burnout, meaninglessness), consistent with COR’s concept of resource caravans and loss spirals [28]. This suggests that protecting this specific keystone resource may be more effective than bolstering peripheral resources like task management skills [29]. The finding corroborates the core tenet of COR theory that sustained resource loss triggers psychological defense, and further reveals, from a systemic perspective, the associative pathways through which resource depletion occurs in high‑pressure clinical environments [30]. As a core dimension of moral distress, the high centrality of personal responsibility suggests that nursing interns commonly internalize patient safety and care quality as personal obligations [26]. When environmental constraints prevent them from fulfilling these obligations, a pronounced perception of resource loss is triggered, thereby accelerating the depletion of emotional and cognitive resources [25,31].

Meanwhile, the strong connectivity of the burnout node further indicates that emotional resource exhaustion does not occur in isolation; rather, it is associated with multiple stressors, including moral distress and task load, forming a densely interconnected associative cluster consistent with a triple depletion process [32–34]. Failure to maintain the patient’s best interest, a subdimension of moral distress directly tied to clinical decision‑making, warrants particular attention given its high centrality [32,35]. This variable reflects the disconnect between ethical ideals and practical constraints that nursing interns routinely face in real‑world clinical settings [34]. Such dissonance may be associated with eroded professional identity and may also be associated with amplified feelings of powerlessness and meaninglessness, thereby being linked to work alienation [33,36].

Comparison with existing network studies, only one prior study [7] has applied network analysis to moral distress in nursing students, finding harm to patient as a central node. Our study extends this by embedding moral distress within a broader system including compassion fatigue and workload. Unlike Xiong et al. [7] personal responsibility emerged as the most central node, a discrepancy that may stem from differences in sample (Chinese vs. international) or measurement instruments. Our bridge node findings (STS, workload) are partially consistent with prior network studies on healthcare workers [13,37], supporting the cross‑construct bridging role of secondary traumatic stress. However, no prior network study has identified self‑evaluation as a bridge node, which is a contribution of this work.

### Bridge nodes and hypothesized intervention priorities

In network analysis, STS, workload, and self-evaluation exhibited the highest bridge strength, indicating that they serve as key bridges linking distinct psychological constructs such as compassion fatigue, workload, and work alienation [38]. This finding provides deeper insight into how multiple psychological processes in nursing interns’ clinical practice interact through specific nodes, thereby forming a negative cycle that sustains alienation [39]. As a core component of compassion fatigue, the strong bridging role of STS highlights the mediating function of emotional resource depletion between moral distress and work alienation [40]: frequent exposure to patient suffering and traumatic events not only directly triggers emotional burnout but may also indirectly intensify moral distress by heightening sensitivity to ethical conflicts, subsequently fostering emotional detachment from work.

The high bridge strength of the workload node suggests that work pressure does not exist in isolation. Rather, it may be associated with further depletion of the psychological resources available to cope with ethical challenges and emotional demands by influencing individuals’ self‑evaluation of their competence [41,42]. Of note, self‑evaluation, a dimension of the NASA‑TLX scale reflecting individuals’perception of their task performance quality, demonstrates a bridging effect that underscores the moderating role of self‑efficacy in the resource conservation process [41]. When nursing interns perceive themselves as incompetent in clinical tasks, this not only may be directly associated with an exacerbated workload experience but may also be indirectly associated with alienation by diminishing their confidence in addressing moral distress and emotional challenges [42].

It is crucial to note, however, that centrality in a cross‑sectional network reflects associative strength and predictability, not causality or confirmed intervention efficacy. The following implications for intervention should therefore be interpreted as hypothesis‑generating priorities derived from the system’s correlational architecture, requiring validation through longitudinal or experimental designs.With this caveat, network analysis uniquely identifies STS as a bridge node, meaning its influence is not isolated within compassion fatigue but spreads across to work alienation and moral distress. Based on this specific systemic role, interventions should prioritize STS reduction (e.g., via trauma‑informed care training) because targeting a bridge node is hypothesized to block multiple negative pathways simultaneously, a prediction that would not emerge from traditional regression models. Similarly, reducing workload perception via task management and time training, and enhancing self‑evaluation through positive feedback and competence support, may help disrupt the negative network’s maintenance mechanism at the intersection of multiple constructs [43].

### Within‑scale density and cross‑construct connections

Strong internal connections were observed both among the compassion fatigue subdimensions and within the moral distress subdimensions, whereas cross-construct connections were concentrated in a few high-strength edges, such as the association between STS and work alienation subdimensions [44]. This structural characteristic provides important evidence for the development of precise and efficient psychological interventions. Traditional internship education often emphasizes stress management or ethical education in a generalized manner, failing to target the truly active “pain points” within the psychological system [45]. This study found strong connections between burnout and secondary traumatic stress within compassion fatigue, as well as between personal responsibility and failure to maintain the patient’s best interest within moral distress, suggesting that these variables may be simultaneously activated during the same clinical event, forming localized reinforcement loops [13,44]. For example, when nursing interns experience emotional exhaustion due to an inability to alleviate patient suffering, their perception of failing to fulfill caregiving responsibilities may also intensify concurrently, with both factors mutually reinforcing each other and accelerating resource depletion [37,46].

Furthermore, the cross‑construct connection between STS and meaninglessness further indicates that emotional trauma may directly erode nursing interns’ identification with the value of nursing work [47]. Therefore, nursing educators should systematically incorporate “contextualized ethical‑emotional integration training” into both pre‑internship and internship phases [48]. For instance, by using high‑fidelity simulation scenarios that allow students to experience the dual pressures of moral distress and emotional impact, and by guiding them through structured reflection to recognize signals of resource depletion and practice self‑regulation strategies [49]. Simultaneously, clinical supervisors should attend to interns’ psychological reactions following traumatic events, providing timely emotional support and meaning reconstruction guidance to prevent the spread of STS toward meaninglessness [50,51]. Such network structure‑based precise interventions hold promise for interrupting the diffusion pathways of resource depletion at an early stage and enhancing nursing students’ psychological resilience [52].

### The dual role of ethical sensitivity

In this study, the three subdimensions of ethical sensitivity primarily exhibited strong within-scale connections in the network, while their cross-construct bridge strength was relatively low [10]. This finding reveals the dual role that ethical sensitivity may play in the psychological system of nursing interns: it serves both as a sensitizer that precipitates moral distress and as a potential resource for preserving professional meaning [10]. On one hand, high ethical sensitivity makes interns more apt to recognize ethical conflicts and patient suffering in clinical practice, potentially leading to earlier and more profound perceptions of resource threat, manifested as latent linkages between moral distress and compassion fatigue nodes [8,53]. This explains why, under identical task load conditions, some interns are more susceptible to entering cycles of resource depletion, their inherent ethical awareness paradoxically becomes an accelerator of resource loss [54].

On the other hand, the absence of strong direct connections between ethical sensitivity nodes and work alienation subdimensions suggests that its direct effect on alienation may be mediated by moral distress and compassion fatigue [27,55]. This offers important implications for nursing education: merely enhancing ethical sensitivity without accompanying training in ethical decision‑making and emotional regulation may inadvertently increase psychological risk for students [56]. Therefore, future educational interventions should move beyond the singular goal of enhancing sensitivity toward a tripartite integration model of sensitivity‑competence‑resilience [27,56]. That is, while strengthening ethical awareness, educators should also enhance students’ capacity to make ethical decisions and manage emotional investment in complex situations through case‑based discussions, role‑playing, and mentor guidance [57]. Furthermore, efforts should be made to help students transform ethical sensitivity into a source of professional meaning [55,58]. For example, by using narrative medicine writing to guide them in reframing clinical ethical challenges as opportunities for professional growth [58].

## Limitations

Although this study provides insights into the formation mechanism of work alienation among nursing interns through network analysis, several limitations should be acknowledged. First, the cross-sectional design collected all data at a single time point. While network analysis revealed conditional dependencies among variables, it cannot infer causal directions or temporal dynamics. Second, the sample was recruited from nursing interns at tertiary hospitals in the Guanzhong region of Shaanxi Province, China. The use of convenience sampling from a single geographic region and specific hospital types substantially limits the external validity of the findings. The results should be considered specific to this context, and claims of broader applicability to other regions, healthcare systems, or cultural settings are not warranted without replication studies. Third, the study relied on self‑reported scales. Although instruments with good reliability and validity were selected, responses may be subject to social desirability bias or common method bias, particularly for sensitive constructs such as moral distress and compassion fatigue. Harman’s one‑factor test suggested common method bias was not severe, but this test is not definitive. Future studies should consider multi‑method validation incorporating qualitative interviews, behavioral observations, or physiological indicators.

Moreover, this study focused on individual psychological variables based on COR theory but did not adequately incorporate the interactive effects of organizational environmental factors or individual trait factors. Finally, this study did not conduct subgroup analyses (e.g., by gender, hospital type, or night shift status) due to the already complex network estimation and the risk of over‑fragmentation of the sample. Future studies with larger, multi‑site samples are needed to test whether the network structure differs across demographic or clinical subgroups. Therefore, future research should employ longitudinal designs, multi‑center sampling, and integrate multi‑level variables to further validate the causality and ecological validity of the network.

## Conclusion

This study employed psychological network analysis to map the associative structure linking task load, moral distress, compassion fatigue, ethical sensitivity, and work alienation among nursing interns. Personal responsibility, burnout, and failure to maintain the patient’s best interest emerged as central hubs, while secondary traumatic stress, task load, and self‑evaluation served as key bridge nodes in the associative network. These findings advance Conservation of Resources theory by demonstrating that work alienation is associated with intersecting resource loss patterns rather than a single stressor. The identified central and bridge nodes offer hypothesis‑generating priority targets for future intervention research, enabling systematic strategies addressing moral distress, compassion fatigue, and self‑evaluation. This study provides preliminary empirical evidence to inform targeted psychological support programs, pending validation in longitudinal and experimental designs.

## Supporting information

S1 Table Demographic characteristics of the study participants (N = 934). This table presents the distribution of demographic and work‑related variables among the 934 participants. Data are shown as frequencies (n) and percentages (%). For the item “Attitude towards the nursing profession”, the two response categories are “Love” and “It is not a love, it is a necessary way to make a living”. Abbreviation: N, total number of participants.
(DOCX)
