# Supplementary material for: Unveiling the psychological network of work alienation among nursing interns: A resource conservation perspective and network analysis
Source: PLoS One. 2026 Jun 17;21(6):e0351867. doi: 10.1371/journal.pone.0351867 (PMC13274885; doi:10.1371/journal.pone.0351867)
Supplement: S1 Table — This table presents the distribution of demographic and work‑related variables among the 934 participants. Data are shown as frequencies (n) and percentages (%). For the item “Attitude towards the nursing profession”, the two response categories are “Love” and “It is not a love, it is a necessary way to make a living”. Abbreviation: N, total number of participants. (DOCX) [file pone.0351867.s001.docx]

Demographic characteristics of the study participants (N = 934)

| items | Category | cases | Proportion（%） |
| --- | --- | --- | --- |
| Gender | male | 148 | 18.5 |
|  | female | 786 | 84.2 |
| Age (years) | ≤20 | 172 | 18.4 |
|  | ＞20 | 762 | 81.6 |
| Education level | Associate degree | 100 | 10.7 |
|  | Bachelor's degree | 834 | 89.3 |
| Are you of Han ethnicity | yes | 887 | 95.0 |
|  | no | 47 | 5.0 |
| Do you have a religious belief | yes | 80 | 8.6 |
|  | no | 854 | 91.4 |
| Place of origin of students | Urban areas | 204 | 21.8 |
|  | Rural areas | 730 | 78.2 |
| Attitude towards the nursing profession | Love | 551 | 59.0 |
|  | it not, it is a necessary way to make a living | 383 | 41.0 |
| Whether the intern worked night shifts | yes | 728 | 77.9 |
|  | no | 206 | 22.1 |
| Do you feel helpless, powerless, and meaningless at work | yes | 461 | 49.4 |
|  | no | 473 | 50.6 |
| Do you feel frustrated at work | yes | 556 | 59.5 |
|  | no | 378 | 40.5 |
| Do you feel consciously tired from work | yes | 622 | 66.6 |
|  | no | 312 | 33.4 |
| Have you received relevant education or training courses (e.g., nursing ethics or moral-related courses) | yes | 853 | 91.3 |
|  | no | 81 | 8.7 |
