# Supplementary material for: Unveiling the psychological network of work alienation among nursing interns: A resource conservation perspective and network analysis
Source: PLoS One. 2026 Jun 17;21(6):e0351867. doi: 10.1371/journal.pone.0351867 (PMC13274885; doi:10.1371/journal.pone.0351867)
Supplement: Attachment — Submitted filename: Response to Reviewers.doc [file pone.0351867.s003.doc]

Title: Unveiling the Psychological Network of Work Alienation Among Nursing Interns: A Resource Conservation Perspective and Network Analysis

ID: PONE-D-26-10273

**Dear Editor and Reviewers,**

We sincerely thank the reviewers for their thoughtful and constructive comments on our manuscript. We have carefully addressed each concern and revised the manuscript accordingly. Below we provide a point‑by‑point response. All modifications have been highlighted in the revised manuscript using track changes.

**Reviewer #1: Thank you for the opportunity to review this manuscript examining work alienation among nursing interns using a psychological network analysis framework. The topic is important and clinically relevant. The manuscript is generally well-structured, and the sample size is adequate. The use of validated instruments and an attempt to move beyond simple linear models are commendable.**

However, several concerns remain regarding the study’s contribution and interpretation:

1. Limited incremental insight relative to existing literature:
While the application of network analysis is technically appropriate, the findings largely confirm well-established relationships (e.g., workload, burnout, moral distress, and compassion fatigue are interrelated and associated with alienation). The manuscript would benefit from a clearer articulation of what new knowledge is gained from the network approach beyond confirming known associations.

***Answer:****We agree and have added explicit statements about the unique advantages of network analysis.*

*In the Introduction (page 3-4, paragraph 4), we now write: “Unlike traditional linear models that presuppose unidirectional causal pathways and require pre‑specified mediator/moderator relationships, network analysis offers two unique advantages: Unlike traditional linear models that presuppose unidirectional causal pathways and require pre‑specified mediator/moderator relationships, network analysis offers two unique advantages: Firstly, it visualizes the complex, bidirectional interconnections among all variables simultaneously without imposing a priori assumptions[14]. Secondly, it quantifies the relative importance of each variable (centrality) and each cross‑construct connection (bridge strength) within the overall system[15]. This approach allows us to move beyond merely confirming whether variables are associated to uncovering the specific structural architecture of resource depletion, potentially identifying novel intervention targets that are not apparent from simple pairwise correlations.”*

*In the Discussion (page 11, paragraph 1), we also added: “While prior research has individually linked moral distress, burnout, and work alienation [25], the network analysis reveals their relative centrality within a unified system. Critically, it demonstrates that ‘personal responsibility’ and ‘failure to maintain best interest’ are not just correlates but occupy the most influential hub positions, a system‑level prioritization that is a key incremental insight over traditional models.”*

2.Interpretation of centrality and bridge metrics:
The identification of “central” and “bridge” nodes is presented as actionable, but the practical implications are not fully justified. In cross-sectional network models, centrality does not imply causality or intervention priority. This limitation should be more explicitly acknowledged, and the discussion should avoid over-interpreting these metrics as targets for intervention.

***Answer:****We have added explicit cautionary statements. In the Discussion (page 18, paragraph 2), we now write: “It is crucial to note, however, that centrality in a cross‑sectional network reflects associative strength and predictability, not causality or confirmed intervention efficacy. The following implications for intervention should therefore be interpreted as hypothesis‑generating priorities derived from the system’s correlational architecture, requiring validation through longitudinal or experimental designs.*

*Similarly, in the Conclusion we replaced “precise intervention targets” with “hypothesis‑generating priority targets for future intervention research”.*

3.Cross-sectional design limits claims of system dynamics:
The manuscript frequently refers to “dynamic interactions,” “cycles,” and “resource depletion pathways.” However, the study design is cross-sectional and cannot support temporal or causal claims. The language throughout the discussion and conclusion should be revised to reflect associative rather than dynamic or causal interpretations.

***Answer:****We have systematically revised the language throughout the manuscript. Key examples:(1)Abstract: changed “result from chronic resource depletion” to “be statistically associated with a pattern of resource‑related variables”; changed “dynamic interactions” to “associative patterns”.(2)Introduction: changed “creating a vicious cycle” to “which, in cross‑sectional data, would appear as a mutually reinforcing associative loop”.(3)Discussion: changed “forming a responsibility‑emotion‑cognition triple depletion loop” to “forming a densely interconnected associative cluster consistent with a triple depletion process”.All such revisions are marked in track changes.*

4. Model justification and methodological clarity:
Although the statistical methods are described, the rationale for selecting this specific network approach is not fully developed. Additional justification for using subscale-level aggregation rather than item-level data, the choice of regularization parameters, and the applicability of resulting edges would strengthen the methodological transparency.
***Answer:****We have added the required details in the Statistical Analysis section (page 6). Specifically: “The hyperparameter gamma (γ) for the EBICglasso was set to 0.5, a common default that balances model fit and parsimony. Subscale‑level aggregation was chosen over item‑level analysis to maintain a stable ratio of parameters to sample size (934 valid cases for 15 nodes) and to improve the reliability of each node as a construct indicator, consistent with prior network studies on similar constructs.”*

5. Sampling limitations:
The use of convenience sampling from a single geographic region limits external validity. While this is acknowledged, the discussion occasionally implies broader applicability (e.g., implications for nursing education more generally). These claims should be moderated.
***Answer:****We have strengthened the limitation statement in the Limitations section (page 20). Now it reads: “The use of convenience sampling from a single geographic region (Guanzhong, Shaanxi) and specific hospital types (tertiary) substantially limits the external validity of the findings. The results should be considered specific to this context, and claims of broader applicability to other regions, healthcare systems, or cultural settings are not warranted without replication studies.”*

6. Intervention implications are generic:
The proposed interventions are reasonable but not clearly derived from the network analysis itself. As written, the recommendations could be made without the current modeling approach. Clarifying how the network findings uniquely inform intervention prioritization would improve the manuscript.
***Answer:****We have revised the intervention section to explicitly link each recommendation to the specific role of the node (central vs. bridge). In the Discussion (page 18, paragraph 3): “Network analysis uniquely identifies STS as a bridge node-meaning its influence is not isolated within compassion fatigue but spreads across to work alienation and moral distress. Based on this specific systemic role, interventions should prioritize STS reduction (e.g., via trauma‑informed care training) because targeting a bridge node is hypothesized to block multiple negative pathways simultaneously, a prediction that would not emerge from traditional regression models.” Similar justifications are added for workload and self‑evaluation.*

7. Language and phrasing:
The manuscript is generally understandable, but some phrasing remains awkward or overly literal (e.g., “this study map the psychological network…”). A final round of language editing would improve readability.

***Answer:****We have corrected the specific error: the conclusion now reads “This study mapped the psychological network…”. We have also performed a full language check and made numerous small improvements to readability throughout the manuscript (please see track changes).*

**Reviewer #2: This study explores the mechanism of work alienation among nursing interns using psychological network analysis, which holds certain significance for nursing education and mental health interventions for interns. The research design is relatively rigorous and the network analysis method is appropriately applied. However, further refinement and polishing are required in terms of research design details, result interpretation, depth of discussion and writing standardization. Major revisions are required before the manuscript can proceed to the subsequent review process.**1. Study Design and ParticipantsThe manuscript fails to supplement the basic demographic characteristics of the valid sample, nor does it explain the potential selection bias of convenience sampling and the corresponding bias control measures. In addition, it is necessary to clarify whether the four tertiary hospitals include general hospitals and specialized hospitals, as this information directly affects the representativeness of the sample.The existing inclusion and exclusion criteria only exclude interns with psychological disorders or severe mental illnesses, but do not formulate clear evaluation criteria and screening methods for "psychological disorders/severe mental illnesses" (e.g., whether standardized scales or clinical diagnoses are adopted). Specific screening procedures need to be supplemented to ensure the homogeneity of the basic psychological status of the research subjects.***Answer:****We have added a new subsection “Participant Characteristics” in the Results (page 7, Table S1 in supplementary materials, and summary in main text). The summary includes: gender (84.2% female), mean age (21.4±1.2), education level (89.3% undergraduate), ethnicity (95% Han), religious belief (8.6%), place of origin (78.2% rural), attitude toward nursing profession (59% “like”), and night shift status (77.9%).We also specified that the four tertiary hospitals were general hospitals, added in the Data Collection section (page 6).Regarding exclusion for psychological disorders, we now explicitly state in the Participants section (page 5): “No standardized diagnostic scales were administered; exclusion was based solely on participant disclosure of a formal diagnosis, which may introduce selection bias.”Selection bias is also acknowledged in the Limitations (page 20): “Although instruments with good reliability and validity were selected, responses may be subject to social desirability bias or common method bias, particularly for sensitive constructs such as moral distress and compassion fatigue.”*

2. MethodologyInsufficient description of network analysis operation details: The manuscript mentions constructing a regularized network model based on partial correlation and adopting the EBICglasso method for regularization, but does not report the specific parameter settings in the analysis process.Inadequate control of common method bias: This study is a cross-sectional survey based on self-reported scales, which is prone to common method bias. However, the manuscript does not mention any common method bias control measures adopted in the research design stage, nor conduct statistical tests for common method bias in the data analysis stage. The corresponding test results and explanations need to be supplemented.***Answer:****We have added the gamma value (γ = 0.5) in the Statistical Analysis section (page 8).For CMB, we added a separate subsection: “Common Method Bias (CMB)” in Methods and reported the Harman’s one‑factor test result in the Results (page 9): “The unrotated factor solution explained 28.6% of the total variance, which is below the recommended threshold of 40%, suggesting that CMB is unlikely to severely distort the correlational results.”*3. ResultsThe manuscript only lists the top three core nodes and top four bridge nodes, but does not provide the specific values of node strength and bridge strength for all nodes, nor explain the statistical significance test methods for the differences in node strength/bridge strength.The manuscript fails to explore the differences in the psychological network structure of work alienation among groups with different demographic characteristics. Subgroup analysis results need to be supplemented to further enrich the research findings and provide a basis for formulating more targeted intervention suggestions.***Answer:****We have added a new Table 2 (page 13) that reports node strength and bridge strength for all 15 subscales with 95% bootstrap confidence intervals. This directly addresses the request for complete numerical values and significance via non‑overlapping CIs.*

*Regarding subgroup analysis, we have now conducted subgroup network comparisons by education, religion, ethnicity, gender, and age. The results are presented in a new Table 3 (page 16) and described in the “Subgroup Network Analysis Results” subsection. The Network Comparison Test showed no statistically significant differences in global network structure across these subgroups (all p > 0.05), although descriptive differences in global strength and top nodes are noted. This addition fulfills the reviewer’s request.*

4. Discussion and Study LimitationsThe manuscript only briefly mentions that the research results verify the core viewpoints of the Conservation of Resources Theory, but does not combine the core nodes and bridge nodes of the network to deeply analyze how they reflect the process of "resource loss - resource protection - resource depletion" among nursing interns. It is necessary to explain the theoretical connotation of the research results in combination with the specific network connection characteristics, so as to further expand the application of the Conservation of Resources Theory in the research field of nursing interns.The manuscript does not compare the research results with previous network analysis studies on work alienation/moral distress/compassion fatigue of nursing staff, nor explain the similarities and differences between the current research results and previous studies and the reasons for the differences. A comparative discussion with existing studies needs to be supplemented.The manuscript proposes intervention strategies such as "ethical support, trauma-informed care, and competence feedback", but does not design targeted strategies combined with the core nodes and bridge nodes of the network, nor explain the implementation paths and expected effects of the strategies. The intervention strategies need to be refined to make them more in line with the clinical practice of nursing interns and have practical operability.Specific and feasible suggestions for improving future research should be put forward for each limitation.***Answer:*** *COR theory integration: We have expanded the theoretical interpretation throughout the Discussion. For example (page 17): “From a Conservation of Resources (COR) perspective, this high centrality indicates that ‘personal responsibility’ functions as a keystone resource. When threatened or lost … consistent with COR’s concept of resource caravans and loss spirals [28]. This suggests that protecting this specific keystone resource may be more effective than bolstering peripheral resources like task management skills [29].”Comparison with prior network studies: We added a paragraph (page 17-18): “Comparison with existing network studies… only one prior study [7] has applied network analysis to moral distress in nursing students, finding harm to patient as a central node. Our study extends this … Our bridge node findings (STS, workload) are partially consistent with prior network studies on healthcare workers [13, 37], supporting the cross‑construct bridging role of secondary traumatic stress. However, no prior network study has identified self‑evaluation as a bridge node, which is a contribution of this work.”Intervention strategies: As also responded to Reviewer #1, we have linked each intervention recommendation directly to the node’s central/bridge role and added hypothesis‑generating language (see response to Comment 1.6).*5. Writing StandardizationUnification of the expression of professional terms and abbreviations: Some terms in the manuscript are used in abbreviated form after their first appearance (e.g., STS, UKA), but there are problems such as individual abbreviations not being defined at their first appearance and inconsistent expression of the same term. A full manuscript check is required to standardize the definition of abbreviations (uniformly marked in the abstract and keywords) and unify the expression of the same term.Supplement missing references and standardize citation formats: Some statements in the manuscript lack the support of corresponding references, and the citation formats of references are not completely unified. High-quality missing references need to be supplemented.Optimize figure and table descriptions: The manuscript mentions Table 1 and Figures 1-3, but the descriptions of the figure and table legends are not detailed enough. The figure and table legends need to be optimized with necessary notes added to ensure that the figures and tables can be understood independently without referring to the main text.***Answer:****We have carefully reviewed the manuscript:(1)All abbreviations (STS, CSat, MDS, NASA‑TLX, etc.) are now defined at first use in the abstract and main text.(2)Reference list has been reformatted and missing information added .*

*(3)Figure legends have been expanded. For example, Figure 1 legend now reads: “Left panel: Regularized partial correlation network of all 16 subscales. Green solid edges = positive conditional correlations, red dashed edges = negative. Edge thickness = absolute correlation strength. Right panel: Node strength centrality (standardized z‑scores) for each node, with error bars representing 95% bootstrap confidence intervals.”Similar detailed legends are added for Figures 2 and 3.*6. Abstract and KeywordsThe abstract is overly lengthy, and the results section only qualitatively describes the core nodes and bridge nodes without reporting key quantitative results (e.g., the specific bridge strength value of STS). It is recommended to streamline the content of the background and methods sections, delete repetitive statements, and supplement core quantitative data in the results section to make the abstract more concise and informative.The current keywords are "nursing interns; work alienation; compassion fatigue; moral distress; ethical sensitivity", lacking the core research method keyword ("psychological network analysis") and the core research variable keyword ("workload") of the study. It is recommended to add the above two keywords and adjust the order of keywords according to the importance of the research content.
***Answer:****We have shortened the abstract to ~266 words, removed redundant background statements, and added key quantitative results: “Bridge strength analysis identified secondary traumatic stress (STS) as having the highest bridge strength (0.43, 95% bootstrap CI [0.31, 0.55]), followed by task load (0.38) and self‑evaluation (0.38).”Keywords have been updated to: “work alienation; compassion fatigue; moral distress; ethical sensitivity; psychological network analysis; workload” (page 3).*

Final remarks: We hope the revised manuscript meets the standard of the esteemed journal. We are looking forward to your positive response. Thank you.
