# Supplementary material for: Unveiling the psychological network of work alienation among nursing interns: A resource conservation perspective and network analysis
Source: PLoS One. 2026 Jun 17;21(6):e0351867. doi: 10.1371/journal.pone.0351867 (PMC13274885; doi:10.1371/journal.pone.0351867)
Supplement: Peer review history

# Peer review: Decision Letter 0

**DOI:** 10.1371/journal.pone.0351867.r001

**Authors:** Ahmed Abdelwahab Ibrahim El-Sayed

19 Apr 2026

PONE-D-26-10273

Unveiling the Psychological Network of Work Alienation Among Nursing Interns: A Resource Conservation Perspective and Network Analysis

PLOS One

Dear Dr.  Guo,

Thank you for submitting your manuscript to PLOS ONE. After careful consideration, we feel that it has merit but does not fully meet PLOS ONE’s publication criteria as it currently stands. Therefore, we invite you to submit a revised version of the manuscript that addresses the points raised during the review process.

Please submit your revised manuscript by Jun 03 2026 11:59PM. If you will need more time than this to complete your revisions, please reply to this message or contact the journal office at plosone@plos.org. When you're ready to submit your revision, log on to https://www.editorialmanager.com/pone/ and select the 'Submissions Needing Revision' folder to locate your manuscript file.

Please include the following items when submitting your revised manuscript:

- A letter that responds to each point raised by the academic editor and reviewer(s). You should upload this letter as a separate file labeled 'Response to Reviewers'.
- A marked-up copy of your manuscript that highlights changes made to the original version. You should upload this as a separate file labeled 'Revised Manuscript with Track Changes'.
- An unmarked version of your revised paper without tracked changes. You should upload this as a separate file labeled 'Manuscript'.

If you would like to make changes to your financial disclosure, please include your updated statement in your cover letter. Guidelines for resubmitting your figure files are available below the reviewer comments at the end of this letter.

If applicable, we recommend that you deposit your laboratory protocols in protocols.io to enhance the reproducibility of your results. Protocols.io assigns your protocol its own identifier (DOI) so that it can be cited independently in the future. For instructions see: https://journals.plos.org/plosone/s/submission-guidelines#loc-laboratory-protocols. Additionally, PLOS ONE offers an option for publishing peer-reviewed Lab Protocol articles, which describe protocols hosted on protocols.io. Read more information on sharing protocols at https://plos.org/protocols?utm_medium=editorial-email&utm_source=authorletters&utm_campaign=protocols.

As the corresponding author, your ORCID iD is verified in the submission system and will appear in the published article. PLOS supports the use of ORCID, and we encourage all coauthors to register for an ORCID iD and use it as well. Please encourage your coauthors to verify their ORCID iD within the submission system before final acceptance, as unverified ORCID iDs will not appear in the published article. *Only*    the individual author can complete the verification step; PLOS staff *cannot*    verify ORCID iDs on behalf of authors.

We look forward to receiving your revised manuscript.

Kind regards,

Ahmed Abdelwahab Ibrahim El-Sayed

Academic Editor

PLOS One

Journal Requirements:

When submitting your revision, we need you to address these additional requirements.

1.Please ensure that your manuscript meets PLOS ONE's style requirements, including those for file naming. The PLOS ONE style templates can be found at

https://journals.plos.org/plosone/s/file?id=wjVg/PLOSOne_formatting_sample_main_body.pdf and

https://journals.plos.org/plosone/s/file?id=ba62/PLOSOne_formatting_sample_title_authors_affiliations.pdf

2. We note that you have indicated that there are restrictions to data sharing for this study. PLOS only allows data to be available upon request if there are legal or ethical restrictions on sharing data publicly. For more information on unacceptable data access restrictions, please see http://journals.plos.org/plosone/s/data-availability#loc-unacceptable-data-access-restrictions.

Before we proceed with your manuscript, please address the following prompts:

a) If there are ethical or legal restrictions on sharing a de-identified data set, please explain them in detail (e.g., data contain potentially identifying or sensitive patient information, data are owned by a third-party organization, etc.) and who has imposed them (e.g., a Research Ethics Committee or Institutional Review Board, etc.). Please also provide contact information for a data access committee, ethics committee, or other institutional body to which data requests may be sent.

b) If there are no restrictions, please upload the minimal anonymized data set necessary to replicate your study findings to a stable, public repository and provide us with the relevant URLs, DOIs, or accession numbers. For a list of recommended repositories, please see

https://journals.plos.org/plosone/s/recommended-repositories. You also have the option of uploading the data as Supporting Information files, but we would recommend depositing data directly to a data repository if possible.

We will update your Data Availability statement on your behalf to reflect the information you provide.

3. In the online submission form you indicate that your data is not available for proprietary reasons and have provided a contact point for accessing this data. Please note that your current contact point is a co-author on this manuscript. According to our Data Policy, the contact point must not be an author on the manuscript and must be an institutional contact, ideally not an individual. Please revise your data statement to a non-author institutional point of contact, such as a data access or ethics committee, and send this to us via return email. Please also include contact information for the third party organization, and please include the full citation of where the data can be found.

4. When completing the data availability statement of the submission form, you indicated that you will make your data available on acceptance. We strongly recommend all authors decide on a data sharing plan before acceptance, as the process can be lengthy and hold up publication timelines. Please note that, though access restrictions are acceptable now, your entire data will need to be made freely accessible if your manuscript is accepted for publication. This policy applies to all data except where public deposition would breach compliance with the protocol approved by your research ethics board. If you are unable to adhere to our open data policy, please kindly revise your statement to explain your reasoning and we will seek the editor's input on an exemption. Please be assured that, once you have provided your new statement, the assessment of your exemption will not hold up the peer review process.

5. Your ethics statement should only appear in the Methods section of your manuscript. If your ethics statement is written in any section besides the Methods, please move it to the Methods section and delete it from any other section. Please ensure that your ethics statement is included in your manuscript, as the ethics statement entered into the online submission form will not be published alongside your manuscript.

6. We note you have included a table to which you do not refer in the text of your manuscript. Please ensure that you refer to Table 1 in your text; if accepted, production will need this reference to link the reader to the Table.

7. Please include captions for your Supporting Information files at the end of your manuscript, and update any in-text citations to match accordingly. Please see our Supporting Information guidelines for more information: http://journals.plos.org/plosone/s/supporting-information.

8. Thank you for providing your underlying data as Supporting Information.

We note that the data set contains text or data that is not in English. Please note that PLOS is an English-language publisher, so we require data sets to be provided in English as well. Please upload an English-language version of your data set.

This will also allow us to determine if your data follows PLOS standards per our Data Availability policy here: https://journals.plos.org/plosone/s/data-availability.

9. If the reviewer comments include a recommendation to cite specific previously published works, please review and evaluate these publications to determine whether they are relevant and should be cited. There is no requirement to cite these works unless the editor has indicated otherwise.

Additional Editor Comments:

Dear Authors,

Thank you for submitting your manuscript. Your submission has been reviewed, and the reviewers have recommended revisions before it can be considered further for publication.

Please address the reviewers’ comments carefully and revise your manuscript accordingly.

We look forward to receiving your revised version.

[Note: HTML markup is below. Please do not edit.]

Reviewer's Responses to Questions

**Comments to the Author**

1. Is the manuscript technically sound, and do the data support the conclusions?

The manuscript must describe a technically sound piece of scientific research with data that supports the conclusions. Experiments must have been conducted rigorously, with appropriate controls, replication, and sample sizes. The conclusions must be drawn appropriately based on the data presented.

Reviewer #1: Yes

Reviewer #2: Yes

**********

2. Has the statistical analysis been performed appropriately and rigorously?

Reviewer #1: Yes

Reviewer #2: Yes

**********

3. Have the authors made all data underlying the findings in their manuscript fully available?

The PLOS Data policy requires authors to make all data underlying the findings described in their manuscript fully available without restriction, with rare exception (please refer to the Data Availability Statement in the manuscript PDF file). The data should be provided as part of the manuscript or its supporting information, or deposited to a public repository. For example, in addition to summary statistics, the data points behind means, medians and variance measures should be available. If there are restrictions on publicly sharing data—e.g. participant privacy or use of data from a third party—those must be specified.

Reviewer #1: Yes

Reviewer #2: No

**********

4. Is the manuscript presented in an intelligible fashion and written in standard English?

PLOS ONE does not copyedit accepted manuscripts, so the language in submitted articles must be clear, correct, and unambiguous. Any typographical or grammatical errors should be corrected at revision, so please note any specific errors here.

Reviewer #1: Yes

Reviewer #2: Yes

**********

5. Review Comments to the Author

Please use the space provided to explain your answers to the questions above. You may also include additional comments for the author, including concerns about dual publication, research ethics, or publication ethics. (Please upload your review as an attachment if it exceeds 20,000 characters)

Reviewer #1: Thank you for the opportunity to review this manuscript examining work alienation among nursing interns using a psychological network analysis framework. The topic is important and clinically relevant. The manuscript is generally well-structured, and the sample size is adequate. The use of validated instruments and an attempt to move beyond simple linear models are commendable.

However, several concerns remain regarding the study’s contribution and interpretation:

1. Limited incremental insight relative to existing literature:

While the application of network analysis is technically appropriate, the findings largely confirm well-established relationships (e.g., workload, burnout, moral distress, and compassion fatigue are interrelated and associated with alienation). The manuscript would benefit from a clearer articulation of what new knowledge is gained from the network approach beyond confirming known associations.

2. Interpretation of centrality and bridge metrics:

The identification of “central” and “bridge” nodes is presented as actionable, but the practical implications are not fully justified. In cross-sectional network models, centrality does not imply causality or intervention priority. This limitation should be more explicitly acknowledged, and the discussion should avoid over-interpreting these metrics as targets for intervention.

3. Cross-sectional design limits claims of system dynamics:

The manuscript frequently refers to “dynamic interactions,” “cycles,” and “resource depletion pathways.” However, the study design is cross-sectional and cannot support temporal or causal claims. The language throughout the discussion and conclusion should be revised to reflect associative rather than dynamic or causal interpretations.

4. Model justification and methodological clarity:

Although the statistical methods are described, the rationale for selecting this specific network approach is not fully developed. Additional justification for using subscale-level aggregation rather than item-level data, the choice of regularization parameters, and the applicability of resulting edges would strengthen the methodological transparency.

5. Sampling limitations:

The use of convenience sampling from a single geographic region limits external validity. While this is acknowledged, the discussion occasionally implies broader applicability (e.g., implications for nursing education more generally). These claims should be moderated.

6. Intervention implications are generic:

The proposed interventions are reasonable but not clearly derived from the network analysis itself. As written, the recommendations could be made without the current modeling approach. Clarifying how the network findings uniquely inform intervention prioritization would improve the manuscript.

7. Language and phrasing:

The manuscript is generally understandable, but some phrasing remains awkward or overly literal (e.g., “this study map the psychological network…”). A final round of language editing would improve readability.

Overall assessment:

This is a technically competent and methodologically transparent study addressing an important topic. However, the contribution is incremental, and the interpretation of network metrics should be more cautious. With revisions to clarify limitations and moderate claims, the manuscript would be suitable for publication.

Reviewer #2: This study explores the mechanism of work alienation among nursing interns using psychological network analysis, which holds certain significance for nursing education and mental health interventions for interns. The research design is relatively rigorous and the network analysis method is appropriately applied. However, further refinement and polishing are required in terms of research design details, result interpretation, depth of discussion and writing standardization. Major revisions are required before the manuscript can proceed to the subsequent review process.

1. Study Design and Participants

The manuscript fails to supplement the basic demographic characteristics of the valid sample, nor does it explain the potential selection bias of convenience sampling and the corresponding bias control measures. In addition, it is necessary to clarify whether the four tertiary hospitals include general hospitals and specialized hospitals, as this information directly affects the representativeness of the sample.

The existing inclusion and exclusion criteria only exclude interns with psychological disorders or severe mental illnesses, but do not formulate clear evaluation criteria and screening methods for "psychological disorders/severe mental illnesses" (e.g., whether standardized scales or clinical diagnoses are adopted). Specific screening procedures need to be supplemented to ensure the homogeneity of the basic psychological status of the research subjects.

2. Methodology

Insufficient description of network analysis operation details: The manuscript mentions constructing a regularized network model based on partial correlation and adopting the EBICglasso method for regularization, but does not report the specific parameter settings in the analysis process.

Inadequate control of common method bias: This study is a cross-sectional survey based on self-reported scales, which is prone to common method bias. However, the manuscript does not mention any common method bias control measures adopted in the research design stage, nor conduct statistical tests for common method bias in the data analysis stage. The corresponding test results and explanations need to be supplemented.

3. Results

The manuscript only lists the top three core nodes and top four bridge nodes, but does not provide the specific values of node strength and bridge strength for all nodes, nor explain the statistical significance test methods for the differences in node strength/bridge strength.

The manuscript fails to explore the differences in the psychological network structure of work alienation among groups with different demographic characteristics. Subgroup analysis results need to be supplemented to further enrich the research findings and provide a basis for formulating more targeted intervention suggestions.

4. Discussion and Study Limitations

The manuscript only briefly mentions that the research results verify the core viewpoints of the Conservation of Resources Theory, but does not combine the core nodes and bridge nodes of the network to deeply analyze how they reflect the process of "resource loss - resource protection - resource depletion" among nursing interns. It is necessary to explain the theoretical connotation of the research results in combination with the specific network connection characteristics, so as to further expand the application of the Conservation of Resources Theory in the research field of nursing interns.

The manuscript does not compare the research results with previous network analysis studies on work alienation/moral distress/compassion fatigue of nursing staff, nor explain the similarities and differences between the current research results and previous studies and the reasons for the differences. A comparative discussion with existing studies needs to be supplemented.

The manuscript proposes intervention strategies such as "ethical support, trauma-informed care, and competence feedback", but does not design targeted strategies combined with the core nodes and bridge nodes of the network, nor explain the implementation paths and expected effects of the strategies. The intervention strategies need to be refined to make them more in line with the clinical practice of nursing interns and have practical operability.

Specific and feasible suggestions for improving future research should be put forward for each limitation.

5. Writing Standardization

Unification of the expression of professional terms and abbreviations: Some terms in the manuscript are used in abbreviated form after their first appearance (e.g., STS, UKA), but there are problems such as individual abbreviations not being defined at their first appearance and inconsistent expression of the same term. A full manuscript check is required to standardize the definition of abbreviations (uniformly marked in the abstract and keywords) and unify the expression of the same term.

Supplement missing references and standardize citation formats: Some statements in the manuscript lack the support of corresponding references, and the citation formats of references are not completely unified. High-quality missing references need to be supplemented.

Optimize figure and table descriptions: The manuscript mentions Table 1 and Figures 1-3, but the descriptions of the figure and table legends are not detailed enough. The figure and table legends need to be optimized with necessary notes added to ensure that the figures and tables can be understood independently without referring to the main text.

6. Abstract and Keywords

The abstract is overly lengthy, and the results section only qualitatively describes the core nodes and bridge nodes without reporting key quantitative results (e.g., the specific bridge strength value of STS). It is recommended to streamline the content of the background and methods sections, delete repetitive statements, and supplement core quantitative data in the results section to make the abstract more concise and informative.

The current keywords are "nursing interns; work alienation; compassion fatigue; moral distress; ethical sensitivity", lacking the core research method keyword ("psychological network analysis") and the core research variable keyword ("workload") of the study. It is recommended to add the above two keywords and adjust the order of keywords according to the importance of the research content.

**********

6. PLOS authors have the option to publish the peer review history of their article (what does this mean?). If published, this will include your full peer review and any attached files.

If you choose “no”, your identity will remain anonymous but your review may still be made public.

**Do you want your identity to be public for this peer review?**    For information about this choice, including consent withdrawal, please see our Privacy Policy.

Reviewer #1: No

Reviewer #2: No

**********

[NOTE: If reviewer comments were submitted as an attachment file, they will be attached to this email and accessible via the submission site. Please log into your account, locate the manuscript record, and check for the action link "View Attachments". If this link does not appear, there are no attachment files.]

To ensure your figures meet our technical requirements, please review our figure guidelines: https://journals.plos.org/plosone/s/figures

You may also use PLOS’s free figure tool, NAAS, to help you prepare publication quality figures: https://journals.plos.org/plosone/s/figures#loc-tools-for-figure-preparation.

NAAS will assess whether your figures meet our technical requirements by comparing each figure against our figure specifications.

# Author comment: Author response to Decision Letter 1

**DOI:** 10.1371/journal.pone.0351867.r002

19 May 2026

Dear Editor and Reviewers,

We sincerely thank the reviewers for their thoughtful and constructive comments on our manuscript. We have carefully addressed each concern and revised the manuscript accordingly. Below we provide a point by point response. All modifications have been highlighted in the revised manuscript using track changes.

Reviewer #1: Thank you for the opportunity to review this manuscript examining work alienation among nursing interns using a psychological network analysis framework. The topic is important and clinically relevant. The manuscript is generally well-structured, and the sample size is adequate. The use of validated instruments and an attempt to move beyond simple linear models are commendable.

However, several concerns remain regarding the study’s contribution and interpretation:

1. Limited incremental insight relative to existing literature:

While the application of network analysis is technically appropriate, the findings largely confirm well-established relationships (e.g., workload, burnout, moral distress, and compassion fatigue are interrelated and associated with alienation). The manuscript would benefit from a clearer articulation of what new knowledge is gained from the network approach beyond confirming known associations.

Answer:We agree and have added explicit statements about the unique advantages of network analysis.

In the Introduction (page 3-4, paragraph 4), we now write: “Unlike traditional linear models that presuppose unidirectional causal pathways and require pre specified mediator/moderator relationships, network analysis offers two unique advantages: Unlike traditional linear models that presuppose unidirectional causal pathways and require pre specified mediator/moderator relationships, network analysis offers two unique advantages: Firstly, it visualizes the complex, bidirectional interconnections among all variables simultaneously without imposing a priori assumptions[14]. Secondly, it quantifies the relative importance of each variable (centrality) and each cross construct connection (bridge strength) within the overall system[15]. This approach allows us to move beyond merely confirming whether variables are associated to uncovering the specific structural architecture of resource depletion, potentially identifying novel intervention targets that are not apparent from simple pairwise correlations.”

In the Discussion (page 11, paragraph 1), we also added: “While prior research has individually linked moral distress, burnout, and work alienation [25], the network analysis reveals their relative centrality within a unified system. Critically, it demonstrates that ‘personal responsibility’ and ‘failure to maintain best interest’ are not just correlates but occupy the most influential hub positions, a system level prioritization that is a key incremental insight over traditional models.”

2.Interpretation of centrality and bridge metrics:

The identification of “central” and “bridge” nodes is presented as actionable, but the practical implications are not fully justified. In cross-sectional network models, centrality does not imply causality or intervention priority. This limitation should be more explicitly acknowledged, and the discussion should avoid over-interpreting these metrics as targets for intervention.

Answer:We have added explicit cautionary statements. In the Discussion (page 18, paragraph 2), we now write: “It is crucial to note, however, that centrality in a cross sectional network reflects associative strength and predictability, not causality or confirmed intervention efficacy. The following implications for intervention should therefore be interpreted as hypothesis generating priorities derived from the system’s correlational architecture, requiring validation through longitudinal or experimental designs.

Similarly, in the Conclusion we replaced “precise intervention targets” with “hypothesis generating priority targets for future intervention research”.

3.Cross-sectional design limits claims of system dynamics:

The manuscript frequently refers to “dynamic interactions,” “cycles,” and “resource depletion pathways.” However, the study design is cross-sectional and cannot support temporal or causal claims. The language throughout the discussion and conclusion should be revised to reflect associative rather than dynamic or causal interpretations.

Answer:We have systematically revised the language throughout the manuscript. Key examples:

(1)Abstract: changed “result from chronic resource depletion” to “be statistically associated with a pattern of resource related variables”; changed “dynamic interactions” to “associative patterns”.

(2)Introduction: changed “creating a vicious cycle” to “which, in cross sectional data, would appear as a mutually reinforcing associative loop”.

(3)Discussion: changed “forming a responsibility emotion cognition triple depletion loop” to “forming a densely interconnected associative cluster consistent with a triple depletion process”.

All such revisions are marked in track changes.

4. Model justification and methodological clarity:

Although the statistical methods are described, the rationale for selecting this specific network approach is not fully developed. Additional justification for using subscale-level aggregation rather than item-level data, the choice of regularization parameters, and the applicability of resulting edges would strengthen the methodological transparency.

Answer:We have added the required details in the Statistical Analysis section (page 6). Specifically: “The hyperparameter gamma (γ) for the EBICglasso was set to 0.5, a common default that balances model fit and parsimony. Subscale level aggregation was chosen over item level analysis to maintain a stable ratio of parameters to sample size (934 valid cases for 15 nodes) and to improve the reliability of each node as a construct indicator, consistent with prior network studies on similar constructs.”

5. Sampling limitations:

The use of convenience sampling from a single geographic region limits external validity. While this is acknowledged, the discussion occasionally implies broader applicability (e.g., implications for nursing education more generally). These claims should be moderated.

Answer:We have strengthened the limitation statement in the Limitations section (page 20). Now it reads: “The use of convenience sampling from a single geographic region (Guanzhong, Shaanxi) and specific hospital types (tertiary) substantially limits the external validity of the findings. The results should be considered specific to this context, and claims of broader applicability to other regions, healthcare systems, or cultural settings are not warranted without replication studies.”

6. Intervention implications are generic:

The proposed interventions are reasonable but not clearly derived from the network analysis itself. As written, the recommendations could be made without the current modeling approach. Clarifying how the network findings uniquely inform intervention prioritization would improve the manuscript.

Answer:We have revised the intervention section to explicitly link each recommendation to the specific role of the node (central vs. bridge). In the Discussion (page 18, paragraph 3): “Network analysis uniquely identifies STS as a bridge node-meaning its influence is not isolated within compassion fatigue but spreads across to work alienation and moral distress. Based on this specific systemic role, interventions should prioritize STS reduction (e.g., via trauma informed care training) because targeting a bridge node is hypothesized to block multiple negative pathways simultaneously, a prediction that would not emerge from traditional regression models.” Similar justifications are added for workload and self evaluation.

7. Language and phrasing:

The manuscript is generally understandable, but some phrasing remains awkward or overly literal (e.g., “this study map the psychological network…”). A final round of language editing would improve readability.

Answer:We have corrected the specific error: the conclusion now reads “This study mapped the psychological network…”. We have also performed a full language check and made numerous small improvements to readability throughout the manuscript (please see track changes).

Reviewer #2: This study explores the mechanism of work alienation among nursing interns using psychological network analysis, which holds certain significance for nursing education and mental health interventions for interns. The research design is relatively rigorous and the network analysis method is appropriately applied. However, further refinement and polishing are required in terms of research design details, result interpretation, depth of discussion and writing standardization. Major revisions are required before the manuscript can proceed to the subsequent review process.

1. Study Design and Participants

The manuscript fails to supplement the basic demographic characteristics of the valid sample, nor does it explain the potential selection bias of convenience sampling and the corresponding bias control measures. In addition, it is necessary to clarify whether the four tertiary hospitals include general hospitals and specialized hospitals, as this information directly affects the representativeness of the sample.

The existing inclusion and exclusion criteria only exclude interns with psychological disorders or severe mental illnesses, but do not formulate clear evaluation criteria and screening methods for "psychological disorders/severe mental illnesses" (e.g., whether standardized scales or clinical diagnoses are adopted). Specific screening procedures need to be supplemented to ensure the homogeneity of the basic psychological status of the research subjects.

Answer:We have added a new subsection “Participant Characteristics” in the Results (page 7, Table S1 in supplementary materials, and summary in main text). The summary includes: gender (84.2% female), mean age (21.4±1.2), education level (89.3% undergraduate), ethnicity (95% Han), religious belief (8.6%), place of origin (78.2% rural), attitude toward nursing profession (59% “like”), and night shift status (77.9%).

We also specified that the four tertiary hospitals were general hospitals, added in the Data Collection section (page 6).

Regarding exclusion for psychological disorders, we now explicitly state in the Participants section (page 5): “No standardized diagnostic scales were administered; exclusion was based solely on participant disclosure of a formal diagnosis, which may introduce selection bias.”

Selection bias is also acknowledged in the Limitations (page 20): “Although instruments with good reliability and validity were selected, responses may be subject to social desirability bias or common method bias, particularly for sensitive constructs such as moral distress and compassion fatigue.”

2.Methodology

Insufficient description of network analysis operation details: The manuscript mentions constructing a regularized network model based on partial correlation and adopting the EBICglasso method for regularization, but does not report the specific parameter settings in the analysis process.

Inadequate control of common method bias: This study is a cross-sectional survey based on self-reported scales, which is prone to common method bias. However, the manuscript does not mention any common method bias control measures adopted in the research design stage, nor conduct statistical tests for common method bias in the data analysis stage. The corresponding test results and explanations need to be supplemented.

Answer:We have added the gamma value (γ = 0.5) in the Statistical Analysis section (page 8).

For CMB, we added a separate subsection: “Common Method Bias (CMB)” in Methods and reported the Harman’s one factor test result in the Results (page 9): “The unrotated factor solution explained 28.6% of the total variance, which is below the recommended threshold of 40%, suggesting that CMB is unlikely to severely distort the correlational results.”

3. Results

The manuscript only lists the top three core nodes and top four bridge nodes, but does not provide the specific values of node strength and bridge strength for all nodes, nor explain the statistical significance test methods for the differences in node strength/bridge strength.

The manuscript fails to explore the differences in the psychological network structure of work alienation among groups with different demographic characteristics. Subgroup analysis results need to be supplemented to further enrich the research findings and provide a basis for formulating more targeted intervention suggestions.

Answer:We have added a new Table 2 (page 13) that reports node strength and bridge strength for all 15 subscales with 95% bootstrap confidence intervals. This directly addresses the request for complete numerical values and significance via non overlapping CIs.

Regarding subgroup analysis, we have now conducted subgroup network comparisons by education, religion, ethnicity, gender, and age. The results are presented in a new Table 3 (page 16) and described in the “Subgroup Network Analysis Results” subsection. The Network Comparison Test showed no statistically significant differences in global network structure across these subgroups (all p > 0.05), although descriptive differences in global strength and top nodes are noted. This addition fulfills the reviewer’s request.

4. Discussion and Study Limitations

The manuscript only briefly mentions that the research results verify the core viewpoints of the Conservation of Resources Theory, but does not combine the core nodes and bridge nodes of the network to deeply analyze how they reflect the process of "resource loss - resource protection - resource depletion" among nursing interns. It is necessary to explain the theoretical connotation of the research results in combination with the specific network connection characteristics, so as to further expand the application of the Conservation of Resources Theory in the research field of nursing interns.

The manuscript does not compare the research results with previous network analysis studies on work alienation/moral distress/compassion fatigue of nursing staff, nor explain the similarities and differences between the current research results and previous studies and the reasons for the differences. A comparative discussion with existing studies needs to be supplemented.

The manuscript proposes intervention strategies such as "ethical support, trauma-informed care, and competence feedback", but does not design targeted strategies combined with the core nodes and bridge nodes of the network, nor explain the implementation paths and expected effects of the strategies. The intervention strategies need to be refined to make them more in line with the clinical practice of nursing interns and have practical operability.

Specific and feasible suggestions for improving future research should be put forward for each limitation.

Answer: COR theory integration: We have expanded the theoretical interpretation throughout the Discussion. For example (page 17): “From a Conservation of Resources (COR) perspective, this high centrality indicates that ‘personal responsibility’ functions as a keystone resource. When threatened or lost … consistent with COR’s concept of resource caravans and loss spirals [28]. This suggests that protecting this specific keystone resource may be more effective than bolstering peripheral resources like task management skills [29].”

Comparison with prior network studies: We added a paragraph (page 17-18): “Comparison with existing network studies… only one prior study [7] has applied network analysis to moral distress in nursing students, finding harm to patient as a central node. Our study extends this … Our bridge node findings (STS, workload) are partially consistent with prior network studies on healthcare workers [13, 37], supporting the cross construct bridging role of secondary traumatic stress. However, no prior network study has identified self evaluation as a bridge node, which is a contribution of this work.”

Intervention strategies: As also respond

Attachment Submitted filename: Response to Reviewers.doc

# Peer review: Decision Letter 1

**DOI:** 10.1371/journal.pone.0351867.r003

**Authors:** Ahmed Abdelwahab Ibrahim El-Sayed

2 Jun 2026

Unveiling the Psychological Network of Work Alienation Among Nursing Interns: A Resource Conservation Perspective and Network Analysis

PONE-D-26-10273R1

Dear Author,

We’re pleased to inform you that your manuscript has been judged scientifically suitable for publication and will be formally accepted for publication once it meets all outstanding technical requirements.

Within one week, you’ll receive an e-mail detailing the required amendments. When these have been addressed, you’ll receive a formal acceptance letter, and your manuscript will be scheduled for publication.

An invoice will be generated when your article is formally accepted. Please note, if your institution has a publishing partnership with PLOS and your article meets the relevant criteria, all or part of your publication costs will be covered. Please make sure your user information is up-to-date by logging into Editorial Manager at Editorial Manager® and clicking the ‘Update My Information' link at the top of the page. For questions related to billing, please contact billing support.

If your institution or institutions have a press office, please notify them about your upcoming paper to help maximize its impact. If they’ll be preparing press materials, please inform our press team as soon as possible—no later than 48 hours after receiving the formal acceptance. Your manuscript will remain under strict press embargo until 2 pm Eastern Time on the date of publication. For more information, please contact onepress@plos.org.

Kind regards,

Ahmed Abdelwahab Ibrahim El-Sayed,

Academic Editor

PLOS One

Additional Editor Comments

Dear Authors,

Thank you for your efforts in revising and improving your manuscript. I am pleased to inform you that your manuscript has been accepted for publication in PLOS ONE in its current form.

Congratulations on this achievement, and thank you for choosing PLOS ONE as the venue for your work.

Reviewers' comments:

Reviewer's Responses to Questions

**Comments to the Author**

1. If the authors have adequately addressed your comments raised in a previous round of review and you feel that this manuscript is now acceptable for publication, you may indicate that here to bypass the “Comments to the Author” section, enter your conflict of interest statement in the “Confidential to Editor” section, and submit your "accept" recommendation.

Reviewer #2: All comments have been addressed

**********

2. Is the manuscript technically sound, and do the data support the conclusions?

The manuscript must describe a technically sound piece of scientific research with data that supports the conclusions. Experiments must have been conducted rigorously, with appropriate controls, replication, and sample sizes. The conclusions must be drawn appropriately based on the data presented.

Reviewer #2: Yes

**********

3. Has the statistical analysis been performed appropriately and rigorously?

Reviewer #2: Yes

**********

4. Have the authors made all data underlying the findings in their manuscript fully available?

The PLOS Data policy requires authors to make all data underlying the findings described in their manuscript fully available without restriction, with rare exception (please refer to the Data Availability Statement in the manuscript PDF file). The data should be provided as part of the manuscript or its supporting information or deposited to a public repository. For example, in addition to summary statistics, the data points behind means, medians, and variance measures should be available. If there are restrictions on publicly sharing data—e.g., participant privacy or use of data from a third party—those must be specified.

Reviewer #2: No

**********

5. Is the manuscript presented in an intelligible fashion and written in standard English?

PLOS ONE does not copyedit accepted manuscripts, so the language in submitted articles must be clear, correct, and unambiguous. Any typographical or grammatical errors should be corrected at revision, so please note any specific errors here.

Reviewer #2: Yes

**********

6. Review Comments to the Author

Please use the space provided to explain your answers to the questions above. You may also include additional comments for the author, including concerns about dual publication, research ethics, or publication ethics. (Please upload your review as an attachment if it exceeds 20,000 characters.)

Reviewer #2: (No Response)

**********

7. PLOS authors have the option to publish the peer review history of their article (what does this mean?). If published, this will include your full peer review and any attached files.

If you choose "no," your identity will remain anonymous, but your review may still be made public.

**Do you want your identity to be public for this peer review?**    For information about this choice, including consent withdrawal, please see our Privacy Policy.

Reviewer #2: No

**********

# Editor report: Acceptance letter

**DOI:** 10.1371/journal.pone.0351867.r004

**Authors:** Ahmed Abdelwahab Ibrahim El-Sayed

PONE-D-26-10273R1

PLOS One

Dear Dr. Guo,

I'm pleased to inform you that your manuscript has been deemed suitable for publication in PLOS One. Congratulations! Your manuscript is now being handed over to our production team.

At this stage, our production department will prepare your paper for publication. This includes ensuring the following:

* All references, tables, and figures are properly cited

* All relevant supporting information is included in the manuscript submission,

* There are no issues that prevent the paper from being properly typeset

You will receive further instructions from the production team, including instructions on how to review your proof when it is ready. Please keep in mind that we are working through a large volume of accepted articles, so please give us a few days to review your paper and let you know the next and final steps.

Lastly, if your institution or institutions have a press office, please let them know about your upcoming paper now to help maximize its impact. If they'll be preparing press materials, please inform our press team within the next 48 hours. Your manuscript will remain under strict press embargo until 2 pm Eastern Time on the date of publication. For more information, please contact onepress@plos.org.

You will receive an invoice from PLOS for your publication fee after your manuscript has reached the completed accept phase. If you receive an email requesting payment before acceptance or for any other service, this may be a phishing scheme. Learn how to identify phishing emails and protect your accounts at https://explore.plos.org/phishing.

If we can help with anything else, please email us at customercare@plos.org.

Thank you for submitting your work to PLOS ONE and supporting open access.

Kind regards,

PLOS ONE Editorial Office Staff

on behalf of

Dr. Ahmed Abdelwahab Ibrahim El-Sayed

Academic Editor

PLOS One
